# Genetic Aetiology of Nonsyndromic Hearing Loss in Moravia-Silesia

**DOI:** 10.3390/medicina54020028

**Published:** 2018-05-04

**Authors:** Pavlina Plevova, Petra Tvrda, Martina Paprskarova, Petra Turska, Barbara Kantorova, Eva Mrazkova, Jana Zapletalova

**Affiliations:** 1 Department of Medical Genetics, University Hospital Ostrava, 17. listopadu 1790, 708 52 Ostrava, Czech Republic; petula.g@centrum.cz (P.Tv.); MartaPaprskarova@seznam.cz (M.P.); petra.turska@seznam.cz (P.Tu.); baska.kantorova@gmail.com (B.K.); 2 Department of Epidemiology and Public Health, Faculty of Medicine, University of Ostrava, Syllabova 19, 703 00 Ostrava, Zábřeh, Czech Republic; eva.mrazkova@centrum.cz; 3 Department of Otorhinolaryngology, Hospital of Havířov, Dělnická 1132/24, 736 01 Havířov, Czech Republic; 4 Department of Medical Biophysics, Faculty of Medicine and Dentistry, Palacky University Olomouc, Hnevotinska 3, 775 15 Olomouc, Czech Republic; ja.zapletalova@upol.cz

**Keywords:** hearing loss, sensorineural, nonsyndromic, genetics

## Abstract

Background and Objective: Hearing loss is the most common sensory deficit in humans. The aim of this study was to clarify the genetic aetiology of nonsyndromic hearing loss in the Moravian-Silesian population of the Czech Republic. Patients and Methods: This study included 200 patients (93 males, 107 females, mean age 16.9 years, ranging from 4 months to 62 years) with nonsyndromic sensorineural hearing loss. We screened all patients for mutations in *GJB2* and the large deletion del(*GJB6*-D13S1830). We performed further screening for additional genes (*SERPINB6*, *TMIE*, *COCH*, *ESPN*, *ACTG1*, *KCNQ4*, and *GJB3*) with Sanger sequencing on a subset of patients that were negative for *GJB2* mutations. Results: We detected biallelic *GJB2* mutations in 44 patients (22%). Among these patients, 63.6%, 9.1% and 2.3% exhibited homozygous c.35delG, p.Trp24*, and p.Met34Thr mutations, respectively. The remaining 25% of these patients exhibited compound heterozygous c.35delG, c.-23+1G>A, p.Trp24*, p.Val37Ile, p.Met34Thr, p.Leu90Pro, c.235delC, c.313_326del14, p.Ser139Asn, and p.Gly147Leu mutations. We found a monoallelic *GJB2* mutation in 12 patients (6.6%). We found no pathogenic mutations in the other tested genes. *Conclusions:* One fifth of our cohort had deafness related to *GJB2* mutations. The del(*GJB6*-D13S1830), *SERPINB6*, *TMIE*, *COCH*, *ESPN*, *ACTG1*, *GJB3*, and *KCNQ4* mutations were infrequently associated with deafness in the Moravian-Silesian population. Therefore, we suggest that del(*GJB6*-D13S1830) testing should be performed only when patients with deafness carry the monoallelic *GJB2* mutation.

## 1. Introduction

Sensorineural hearing loss can be caused by genetic or exogenous factors. Nonsyndromic hearing loss is the most common type of genetically determined disorder [1]. The most frequent cause of this disease worldwide is a biallelic mutation in the *GJB2* gene, which encodes the gap junction protein, connexin 26, expressed in the supporting cells of the cochlea [2]. Collectively, biallelic *GJB2* mutations explained 15–20% of hearing loss cases, in some studies [3,4]; in other studies, they explained a much higher proportion of *GJB2-*associated hearing loss (30–53%) [5,6,7]. In fact, c.35delG *GJB2* homozygosity was cited as the cause of 63–94% of congenital deafness in the European population [1,8,9,10].

Seeman et al. studied *GJB2*-related hearing loss in the Czech population [11]. They studied the spectrum and frequencies of *GJB2* mutations among 156 unrelated patients with congenital deafness. Biallelic and monoallelic mutations were detected in about 38% and 10% of those patients, respectively. The c.35delG, p.Trp24*, and c.313del14 mutations accounted for 83%, 10% and 4% of the detected pathogenic alleles, respectively. Other mutations detected in their cohort included p.Leu90Pro, p.Lys15Thr, p.Glu147Lys, and p.delGlu120. About 29% and 4% of all patients were homozygous for c.35delG and p.Trp24* mutations, respectively, and 5% carried compound heterozygous pathogenic mutations that included the c.35delG mutation. The p.Trp24* mutation was found exclusively in Romani patients. Seeman et al. showed that testing for only the three most common mutations could detect over 96% of all pathogenic alleles in the Czech Republic [11].

Previous studies have reported a relatively high frequency of the c.-23+1G>A donor splice-site mutation in *GJB2* intron 1 (previously described as IVS1+1G>A) among Czech, Turkish, and Hungarian patients heterozygous for a *GJB2* coding-region mutation [12,13,14]. Seeman and Sakmaryova later estimated that this splice-site mutation was present in about 4% of pathogenic *GJB2* mutations in Czech patients with hearing loss. They suggested that a similar frequency might be expected in other Central European or Slavic populations. Testing for this mutation explained deafness in 45% of Czech patients with *GJB2* monoallelic mutations [12].

Some patients with hearing loss carry compound heterozygous mutations, where a mutation in the *GJB2* gene is combined with the del(*GJB6*-D13S1830) mutation in *GJB6* gene. The *GJB6* gene encodes connexin 30, another protein expressed in mammalian cochlea [5,8,15]. Currently, evidence from human subjects and mice models has strongly suggested that the del(*GJB6*-D13S1830) mutation eliminated a putative *cis*-regulatory element of *GJB2* located within the deleted region [16]. This mutation is frequent in western European, Israeli, and Brazilian populations, but less frequent in Belgian and Australian populations [5,8]. In the Czech Republic, only one case has been reported of a compound heterozygosity that involved *GJB2* and the del(*GJB6*-D13S1830) mutation [17].

The aims of the present study were (1) to determine the prevalence and types of *GJB2* mutations in a cohort of patients with hearing impairments from the Moravian-Silesian population in the Czech Republic; and (2) to determine the prevalence of mutations in seven additional genes, *SERPINB6*, *TMIE*, *ESPN*, *COCH*, *ACTG1*, *KCNQ4*, and *GJB3*, which we selected because they were small in size and amenable to screening.

## 2. Materials and Methods

This study included 200 unrelated patients with nonsyndromic sensorineural hearing loss. To avoid missing a concurrent genetic cause of the disease, we did not impose selection criteria related to patient immaturity, prenatal infection, perinatal asphyxia, or ototoxic drugs. All patients were referred for a bilateral sensorineural hearing impairment to the Department of Genetics, University Hospital of Ostrava, from otorhinolaryngology departments in the Moravian-Silesian region of the Czech Republic.

The severity of hearing loss was classified according to the World Health Organization grades of hearing impairment, as follows: mild (26–40 dB), moderate (41–60 dB), severe (61–80 dB), or profound (81 dB or greater).

Genomic DNA was isolated from peripheral blood with a DNA extraction instrument. We performed bidirectional Sanger sequencing of the coding sequence (i.e., exon 2), the intron-exon boundaries of the *GJB2* gene, and the noncoding region of *GJB2* exon 1, which included the location of c.-23+1G>A splice-site mutation. Sequencing was performed with the Big Dye Terminator Cycle Sequencing Detection Kit v.3.1 and an ABI 3130 genetic analyser (Applied Biosystems, Foster City, CA, USA) according to the manufacturers’ instructions. We detected the del(*GJB6*-D13S1830) mutation using polymerase chain reaction (PCR) and subsequent electrophoresis, as described by del Castillo [5,15].

In selected patients that lacked *GJB2* and *GJB6* mutations, we performed PCR amplification and bidirectional Sanger sequencing of the coding regions and intron-exon boundaries of the *SERPINB6* (NM_001271823.1, *n* = 13), *TMIE* (NM_147196.2, *n* = 13), *COCH* (NM_004086.2, *n* = 13), *ESPN* (NM_031475.2, *n* = 37), *ACTG1* (NM_001199954.1, *n* = 37), *KCNQ4* (NM_004700.3, *n* = 37), and *GJB3* (NM_024009.2, *n* = 37) genes, as described above. The characteristics of hearing loss related to the tested genes and the characteristics of the tested patients are shown in Table 1.

For patients with *GJB2* pathogenic mutations, we also performed parental genotyping with bidirectional Sanger sequencing. Finally, we included a control group of 270 individuals with normal hearing. We screened the control group for the c.337C>T, p. Arg113Cys *ESPN* variant with custom HybProbes (Roche, Basel, Switzerland) in a melting curve analysis. In a control group of 170 individuals with normal hearing, we also studied the frequency of the c.1797_1808delCCCACCGCCGCC *ESPN* variant with PCR, followed by electrophoresis on a 5% polyacrylamide gel.

### 2.1. Statistical Analysis

Qualitative data are described as the frequency and percentage. Quantitative data are expressed as the mean and range. Patients were divided into subgroups stratified by the age of hearing loss onset and by the severity of hearing loss. We performed the Fisher’s exact test and Bonferroni correction to compare patient groups for differences in the *GJB2* mutation detection rates; the numbers of patients with unaffected parents, but with a sibling with hearing loss; and the numbers of patients with a pedigree suggestive of autosomal dominant inheritance. Data were analysed with IBM SPSS Statistics, version 20 (IBM Corporation, Armonk, NY, USA). *p*-values less than 0.05 were considered statistically significant.

### 2.2. Ethical Approval

All individuals included in this study provided written informed consent before study participation. The study was conducted in accordance with the Declaration of Helsinki, and the protocol was approved by the Ethics Committee of the University Hospital Ostrava (Project identification code No. 1113/2010).

## 3. Results

The study cohort of 200 unrelated index patients included 93 males and 107 females, with a mean age of 16.9 years (ranging from 4 months–62 years). Patient were divided into subgroups stratified by the age of hearing loss onset and the severity of hearing loss (Table 2). Eighteen (9%) of the patients were of Romani origin, and there were no other subpopulations. Twenty-two patients (11%) had unaffected parents and a sibling with hearing loss, which suggested an autosomal recessive inheritance. The pedigrees of 13 patients (7%) suggested an autosomal dominant inheritance (Table 2). Significantly higher proportions of patients with pedigrees suggestive of autosomal dominant inheritance were found in the late onset groups (both childhood and adulthood) than in the early prelingual onset group (*p* = 0.0001 and *p* = 0.042, respectively) and in the moderate hearing loss group compared to the severe (*p* = 0.030) and profound (*p* = 0.001) hearing loss groups.

Biallelic pathogenic *GJB2* mutations that explained hearing loss were found in 44 patients (22%) (Table 2 and Table 3). A single pathogenic *GJB2* mutation was found in 12 patients (6%; Table 2 and Table 4). Biallelic and monoallelic *GJB2* mutations were found in five (27.8%) and four (22.2%) of the 18 Romani patients, respectively. The p.Trp24* mutation was detected in the Romanies, and only one patient was compound heterozygous for c.35delG and p.Trp24* mutations. We applied Fisher’s exact post-hoc test with the Bonferroni correction to evaluate the significance of differences in mutation rates between groups. We found significantly more biallelic *GJB2* mutations in the early prelingual onset group compared to the late childhood onset group (*p* = 0.001); in the severe hearing loss group compared to the mild (*p* = 0.004) and moderate (*p* = 0.046) hearing loss groups; and in the profound hearing loss group compared to the mild hearing loss group (*p* = 0.0497).

The delta(*GJB6*-D13S1830) mutation was not detected in any patient. No pathogenic mutation was found in the *SERPINB6*, *TMIE*, *COCH*, *ESPN*, *ACTG1*, *KCNQ4*, or *GJB3* genes. In the *ESPN* gene, two variants were found in two unrelated patients. Both patients were heterozygous carriers, one carried c.337C>T, p.Arg113Cys (rs143577178) and the other carried c.1797_1808delCCCACCGCCGCC, p.Pro600_Pro603del. These variants were inherited from a parent without hearing loss. The Polyphen score was 1.00 for the c.337C>T missense variant, and this variant was not found in any of the 270 control individuals. The c.1797_1808delCCCACCGCCGCC variant was found in five out of 170 control individuals (2.9%).

## 4. Discussion

The present study was designed to determine the prevalence and types of *GJB2* mutations in a cohort of patients with hearing impairments from the Moravian-Silesian population of the Czech Republic. We also aimed to determine the prevalence of mutations in seven additional genes: *SERPINB6*, *TMIE*, *ESPN*, *COCH*, *ACTG1*, *KCNQ*4, and *GJB3*, which were chosen because they were small and amenable to screening. Our results showed that biallelic pathogenic *GJB2* mutations explained the hearing defects in 22% of patients. Homozygous c.35delG, p.Trp24*, and p.Met34Thr mutations were identified in 63.6%, 9.1%, and 2.3% of patients, respectively. No pathogenic mutation was found in the *SERPINB6*, *TMIE*, *COCH*, *ESPN*, *ACTG1*, *KCNQ4*, or *GJB3* genes.

About 95% of the 10.6 million inhabitants of the Czech Republic are ethnically and linguistically Czech. They are descendants of the Slavic people from the Black Sea-Carpathian region, who settled in Bohemia, Moravia and parts of present-day Austria in the 6th century AD [18]. Other ethnic groups include, primarily, Ukrainians, Slovaks, Vietnamese, Romani, Poles, Germans, and Hungarians [19]. Our patient cohort included only Czech and Romani patients.

In about one-fifth of our cohort, hearing loss was explained by biallelic *GJB2* gene mutations. This detection rate was lower than the 38% reported by Seeman et al. for the Czech population [11]. However, Seeman et al. excluded patients with the following conditions: post-lingual hearing loss, clearly acquired aetiology of deafness, clearly dominant inheritance in three or more subsequent generations, and a known genetic syndrome. About 11% of their patients had an affected sibling, which suggested autosomal recessive inheritance [11]. Del Castillo et al. reported 30% biallellic and 10% monoallelic *GJB2* mutation rates in 422 patients with hearing loss. Nearly half of those families included at least two siblings [5]. In our study, 11% of patients had unaffected parents and a sibling affected by hearing loss; i.e., comparable to the study by Seeman et al. [11]. However, we did not impose patient selection criteria, because we wanted to avoid missing concurrent genetic causes of the disease; this difference in study design might explain our lower detection rate, although it was comparable to other studies [3,4].

All the mutations we detected were previously reported. Consistent with other studies, we found that the most common pathogenic genotype was homozygous c.35delG, which was detected in 64% of patients with biallelic *GJB2* mutations [1,8,9,10]. Individuals with the biallelic c.35delG *GJB2* mutation mainly had profound or severe hearing impairments with a prelingual onset. In only three patients, the phenotype was milder; however, there is currently no explanation for this variable expression.

We found that the *GJB2* biallelic mutation detection rate was significantly higher in the early prelingual subgroup and in the severe or profound hearing loss subgroups compared to the other subgroups. This finding was consistent with the finding that homozygous c.35delG was the most frequent genotype. It was previously shown that hearing loss in individuals with biallelic *GJB2* mutations ranged from mild to profound, and it was most commonly nonprogressive [1]. Moreover, hearing loss in individuals homozygous for the c.35delG mutation was significantly more severe than that in individuals heterozygous for compound c.35delG/non-c.35delG genotypes [1]. 

The p.Trp24* mutation (also known as p.W24X) is prevalent in the Romani population. In our study, it was detected exclusively in Romani patients. We found a large number of patients with hearing loss that could be explained by *GJB2* mutations in this population, due to the high degree of consanguinity. Furthermore, patients with deafness frequently carried the p.Trp24* mutation in studies conducted in India and in Romani populations of different European countries [20,21,22].

Our results did not indicate a high prevalence of the c.-23+1G>A donor splice-site *GJB2* mutation in the Moravian-Silesian population. Indeed, it was found in only two members of one family. Some studies indicated that the p.Val37Ile and p.Met34Thr missense variants were benign polymorphisms [3,23]. Results of other studies supported their pathogenicity and implicated their association with a mild-to-moderate hearing impairment [1,24,25]. We detected one compound c.35delG and p.Val37Ile heterozygous individual with a severe prelingual hearing disorder.

We believe that, in the majority of patients, monoallelic *GJB2* mutations were found at random and the hearing losses were due to different causes. In our study, four of the 12 patients with monoallelic mutations were Romani, and there was a high prevalence of the p.Trp24* in that population. One patient with prelingual profound deafness was heterozygous for p.Met34Thr, but that genotype did not correspond to the phenotype.

The del(*GJB6*-D13S1830) mutation was not detected in our cohort. Our results, consistent with data provided by Seeman et al. [17], suggested that this deletion was not frequent in Slavic populations. Therefore, we recommend that del(*GJB6*-D13S1830) testing should be performed only in patients with deafness that carry a monoallelic *GJB2* mutation.

In some patients, the *GJB2* mutations did not explain their hearing loss. We selected those patients for further study. We chose to screen for small genes with fewer than 15 exons, which were known to display either autosomal recessive or autosomal dominant inheritance, and which had not been tested in the Czech population at the start of the present study. We found no clearly pathogenic mutation in the *SERPINB6* or *TMIE* genes in children with an affected sibling. Moreover, no pathogenic mutation was found in the *ACTG1*, *KCNQ4*, *GJB3*, or *COCH* genes in patients with late onset hearing loss and autosomal dominant pedigrees or in patients with sporadic hearing loss. Only one previous study, by Markova et al., has addressed the aetiology of non-*GJB2*-related hearing loss in the Czech Republic. They studied a panel of small autosomal recessive genes with less than 10 coding exons, including *CABP2*, *CIB2*, *PJVK/DFNB59*, *GJB3*, *ILDR1*, *LHFPL5*, *LRTOMT*, and *TMIE*, in 45–66 Czech patients. One patient displayed compound heterozygosity for two previously described pathogenic variants of the *LHFPL5* gene. In five patients, they found five rare, heterozygous variants—predicted to be pathogenic—in the *CABP2*, *ILDR1*, *LHFPL5* and *LRTOMT* genes [26].

We found two heterozygous patients that carried variants of the *ESPN* gene. *ESPN* gene mutations are inherited in an autosomal recessive manner, and in our patients, the variants were inherited from one of the parents without hearing loss. One variant, c.1797_1808delCCCACCGCCGCC, was found in 2.9% of the control population in our study. This variant was registered under rs548962140 in the Ensembl database [27]. Its frequency in European non-Finnish and Finnish populations was 1.1% and 1.8%, respectively [27]. Thus, this variant is a polymorphism and must be classified as benign. The other variant, c.337C>T, was registered under rs143577178 as a variant of uncertain significance in the Ensembl database. It had a minor allele frequency of 0.1% [27].

One major limitation of this study was the method of selecting genes other than *GJB2* for testing. We chose to test genes that were small in size. In particular, we did not test the most frequently mutated genes in patients with autosomal recessive hearing loss, such as *SLC26A4*, *USH2A*, and *MYO7A*, because their large size would have made Sanger sequencing labour-intensive. We omitted testing other important genes, like *CDH23*, *PCDH15*, *TMPRSS3*, or *WFS1* for similar reasons. Another limitation was the small sample size, particularly the small sizes of the subgroups selected for testing the other genes. Pathogenic mutations in these genes typically cause hearing loss in isolated families. Therefore, testing a larger group of patients could have increased the detection rate.

## 5. Conclusions

We studied gene mutations in a group of unselected patients with hearing loss that developed in infancy, childhood, or adulthood. We found that *GJB2* gene mutations frequently caused hearing loss in the Moravian-Silesian population, and their distribution was similar to that reported in other Caucasian European populations. The low frequency of the del(*GJB6*-D13S1830) deletion in this population indicated that it was not a common cause of deafness. Therefore, we recommend that del(*GJB6*-D13S1830) testing should be performed only in patients with deafness that carry a monoallelic *GJB2* mutation. Although we tested small numbers of patients for *SERPINB6*, *TMIE*, *COCH*, *ACTG1*, *KCNQ4*, and *GJB3* gene mutations, our results suggested that none of these genes was responsible for hearing loss in a substantial proportion of patients.

## Figures and Tables

**Table 1:** Characteristics of genes tested in this study (other than *GJB2* or *GJB6* genes) and patient characteristics.

Gene	Exons/Coding Exons, *N*	Classification of Hearing Loss	Type of Inheritance and Hearing Loss Characteristics	Characteristics of the Tested Patients
***SERPINB6***	8/7	DFNB91	AR, congenital or late onset, progressive	13 pts with a sibling affected with hearing loss, including: 11 pts with early onset prelingual hearing loss and 2 pts with late onset hearing loss in childhood
***TMIE***	4/4	DFNB6	AR, congenital severe to profound	13 pts with a sibling affected with hearing loss, including: 11 pts with early onset prelingual hearing loss and 2 pts with late onset hearing loss in childhood
***COCH***	12/11	DFNA9	AD, adult onset, progressive	13 pts with late onset hearing loss, including: 4 pts with childhood onset and 9 pts with adulthood onset. Pedigrees suggested AD inheritance (*n* = 3) or sporadic (*n* = 10)
***ESPN***	13/13	DFNB36	AR, congenital severe to profound; AD, late onset progressive	37 pts with late onset hearing loss, including: 28 pts with childhood onset and 9 pts with adulthood onset. Pedigrees suggested AD inheritance (*n* = 10) or sporadic (*n* = 27)
***ACTG1***	6/5	DFNA20/26	AD, late onset, progressive	37 pts with late onset hearing loss, including: 28 pts with childhood onset and 9 pts with adulthood onset. Pedigrees suggested AD inheritance (*n* = 10) or sporadic (*n* = 27)
***KCNQ4***	14/14	DFNA2A	AD, late onset, progressive	37 pts with late onset hearing loss, including: 28 pts with childhood onset and 9 pts with adulthood onset. Pedigrees suggested AD inheritance (*n* = 10) or sporadic (*n* = 27)
***GJB3***	2/1	DFNA2B	AD, adult onset, progressive; digenic AR (*GJB2/GJB3*), congenital	37 pts with late onset hearing loss, including: 28 pts with childhood onset and 9 pts with adulthood onset. Pedigrees suggested AD inheritance (*n* = 10) or sporadic (*n* = 27)

Abbreviations: AR, autosomal recessive; AD, autosomal dominant; pts, patients.

**Table 2:** Rates of *GJB2* mutations in hearing loss onset and severity subgroups.

Hearing Loss Groups and Subgroups	*N* (% of Total)	Pts with Unaffected Parents and a Sibling with Hearing Loss/pt Pedigree Suggestive of AD Inheritance, *N* (% of Subgroup)	Pts with Biallelic *GJB2* Mutations, *N* (% of Subgroup)	Pts with Monoallelic *GJB2* Mutations, *N* (% of Subgroup)	Pts without *GJB2* Mutations, *N* (% of Subgroup)
**Onset**					
Early prelingual	104 (52)	13 (13)/0 (0)	35 (34)	4 (4)	65 (62)
Early postlingual	42 (21)	5 (12)/2 (5)	6 (14)	4 (10)	32 (76)
Late, in childhood	44 (22)	4 (9)/9 (21)	2 (5)	4 (9)	38 (86)
Late, in adulthood	10 (5)	0 (0)/2 (20)	1 (10)	0 (0)	9 (90)
Total number	200 (100)	22 (11)/13 (7)	44 (22)	12 (6)	144 (72)
Fisher’s exact test	-	0.831/< 0.0001	0.0002	0.359	0.011
**Severity**					
Mild	45 (23)	3 (7)/2 (4)	3 (7)	3 (7)	39 (86)
Moderate	33 (17)	3 (9)/8 (24)	3 (9)	5 (15)	25 (76)
Severe	68 (34)	9 (13)/3 (4)	23 (34)	1 (1)	44 (65)
Profound	54 (27)	7 (13)/0 (0)	15 (28)	3 (6)	36 (66)
Total number	200 (100)	22 (11)/13 (7)	44 (22)	12 (6)	144 (72)
Fisher’s exact test	-	0.698/0.0002	0.001	0.053	0.055

Early onset, age ≤ 6 years; late onset, age > 6 years. Abbreviations: pts, patients; AD, autosomal dominant.

**Table 3:** Pathogenic biallelic mutations in the *GJB2* gene.

HGVS, Coding Level	HGVS, Protein Level	*N* (%) (*n* = 200)	% Biallelic *GJB2* Mutations (*n* = 44)	Grade and Type of Hearing Loss ^a^
**Biallelic**				
c.[-23+1G>A];[35delG]	p.[?];[Gly12Valfs*2]	1 (0.5)	2.3	Profound
c.[-23+1G>A];[101T>C]	p.[?];[Met34Thr]	1 (0.5)	2.3	Mild PostL
c.[35delG];[35delG]	p.[Gly12Valfs*2];[Gly12Valfs*2]	28 (14)	63.6	13 profound, 13 severe, 1 moderate, 1 mild
c.[35delG];[71G>T]	p.[Gly12Valfs*2];[Trp24*]	1 (0.5)	2.3	Profound
c.[35delG];[101T>C]	p.[Gly12Valfs*2];[Met34Thr]	1 (0.5)	2.3	Moderate PostL
c.[35delG];[109G>A]	p.[Gly12Valfs*2];[Val37Ile]	1 (0.5)	2.3	Severe
c.[35delG];[235delC]	p.[Gly12Valfs*2];[Leu79CysfsTer3]	1 (0.5)	2.3	Severe
c.[35delG];[269T>C]	p.[Gly12Valfs*2];[Leu90Pro]	1 (0.5)	2.3	Moderate PostL
c.[35delG];[313_326del14]	p.[Gly12Valfs*2];[Lys105Glyfs]	1 (0.5)	2.3	Severe
c.[35delG];[439G>A]	p.[Gly12Valfs*2];[Glu147Lys]	1 (0.5)	2.3	Severe
c.[71G>T];[71G>T]	p.[Trp24*];[Trp24*]	4 (2)	9.1	Severe
c.[101T>C];[101T>C]	p.[Met34Thr];[Met34Thr]	1 (0.5)	2.3	Mild PostL
c.[250G>A];[416G>A]	p.[Val84Met];[Ser139Asn]	1 (0.5)	2.3	Severe
c.[313_326del14];[269T>C]	p.[Lys105Glyfs];[Leu90Pro]	1 (0.5)	2.3	Severe PostL
Total number		44 (21)		

^a^ When not indicated, the type of hearing loss was prelingual. Abbreviations: HGVS, Human Genome Variation Society nomenclature; pts, patients; PostL, postlingual.

**Table 4:** Pathogenic monoallelic mutations in the *GJB2* gene.

HGVS, Coding Level	HGVS, Protein Level	*N* (%) (*n* = 200)	Grade and Type of Hearing Loss ^a^
c.[35delG];[=]	p.[Gly12Valfs*2];[=]	3 (1.5)	1 profound, 1 severe, 1 moderate
c.[71G>T];[=]	p.[Trp24*];[=]	3 (1.5)	1 severe, 2 moderate
c.[109G>A];[=]	p.[Val37Ile];[=]	4 (2)	2 moderate, 2 mild PostL
c.[101T>C];[=]	p.[Met34Thr];[=]	2 (1)	1 profound, 1 mild PostL
Total number		12 (6)	

^a^ When not indicated, the type of hearing loss was prelingual. Abbreviations: HGVS, Human Genome Variation Society nomenclature; pts, patients; PostL, postlingual.
